# High-Copy Overexpression Screening Reveals *PDR5* as the Main Doxorubicin Resistance Gene in Yeast

**DOI:** 10.1371/journal.pone.0145108

**Published:** 2015-12-21

**Authors:** Ayse Banu Demir, Ahmet Koc

**Affiliations:** 1 Izmir Institute of Technology, Department of Molecular Biology and Genetics, Urla, Izmir, Turkey; 2 Dokuz Eylul University, Institute of Oncology, Department of Basic Oncology, Izmir, Turkey; Texas A&M University, UNITED STATES

## Abstract

Doxorubicin is one of the most potent anticancer drugs used in the treatment of various cancer types. The efficacy of doxorubicin is influenced by the drug resistance mechanisms and its cytotoxicity. In this study, we performed a high-copy screening analysis to find genes that play a role in doxorubicin resistance and found several genes (*CUE5*, *AKL1*, *CAN1*, *YHR177W* and *PDR5*) that provide resistance. Among these genes, overexpression of *PDR5* provided a remarkable resistance, and deletion of it significantly rendered the tolerance level for the drug. Q-PCR analyses suggested that transcriptional regulation of these genes was not dependent on doxorubicin treatment. Additionally, we profiled the global expression pattern of cells in response to doxorubicin treatment and highlighted the genes and pathways that are important in doxorubicin tolerance/toxicity. Our results suggest that many efflux pumps and DNA metabolism genes are upregulated by the drug and required for doxorubicin tolerance.

## Introduction

Doxorubicin is an anthracycline with a strong anticancer activity. It exerts its effects through different mechanisms, such as intercalation into DNA and inhibition of DNA/RNA biosynthesis, formation of free radicals, inhibition of topoisomerase II, changing membrane properties and inhibition of RNA helicase [1,2]. Unfortunately, these different modes of actions for doxorubicin bring along serious side effects. The most notable one is its cardiotoxicity [3]. Doxorubicin leads to iron accumulation and ROS production, which eventually damages mitochondria and leads to cardiac problems [4].

Multi-drug resistance (MDR) is believed to be an important cause of the treatment failure in metastatic cancer patients [5]. The efflux of the chemotherapeutics by membrane transporters is the main mechanism leading to MDR. Even though mechanisms of MDR have not been revealed exclusively, evading drug resistance and controlling MDR, have been a great issue in chemotherapy.

The mechanisms of doxorubicin resistance have been studied both in yeast and mammalian cells. In mammals, resistance mechanisms include primarily drug efflux from the cell via upregulation of the P-glycoprotein [6], multidrug resistance protein (MRP) [7], anthracycline resistance associated protein MRP6 [8], breast cancer resistance protein (BCRP) [9], and lung resistance-related protein (LRP) [10]. Additionally, changes in the topoisomerase II expression [11], overexpression of glutathione S-transferase (GST) [12], and changes in ERK1/ERK2 proteins [13] provide resistance to doxorubicin.

The budding yeast *S*. *cerevisiae* is a valuable model to identify doxorubicin resistance and sensitivity genes. Screening of deletion collection strains for non-essential genes and studies with specific genes have revealed many genes and pathways in doxorubicin tolerance in yeast [14,15]. These include Ssl2 protein [16], Bsd2 protein [17], SUMO pathway [18], nascent polypeptide-associated complex activity in ribosomes [19], extracellular signal-regulated kinases *ERK1* and *ERK2* [13], endocytic Ark/Prk kinase [20], nitrogen permease regulator 2 (*Npr2*) [21], cytochrome oxidase subunit IV gene [22], and overexpression of *CLN1*, *CLN2* and *ERG13* [23]. Additionally, checkpoint and recombination functions in G1 and early S phase [14], as well as several proteins involved in DNA repair, RNA metabolism, chromatin remodeling, amino acid metabolism, and heat shock response [15], play roles in doxorubicin resistance.

Identification of new genes that play role in cancer drug resistance may provide further prognostic information, which in turn may help to improve the development of new chemotherapeutic agents and increase efficacy of chemotherapeutics. In this study, we intended to identify doxorubicin resistance mechanisms by performing a high copy genomic DNA library screening in the presence of doxorubicin. Several new genes were found to cause resistance against high level of doxorubicin (500μM). Among these genes, *PDR5* had the most remarkable effect on doxorubicin resistance. We also profiled the expression pattern of yeast genome for doxorubicin treatment and highlighted the paths that played roles in resistance and detoxification for this drug.

## Materials and Methods

### Yeast strains, cell growth and plasmids

The BY4741 *(*MATa, *his3*Δ*1 leu2*Δ*0 met15*Δ*0 ura3*Δ*0)* and BY4743 (MATa, *his3*Δ*1/his3*Δ*1 leu2*Δ*0/leu2*Δ*0 LYS2/lys2*Δ*0 met15*Δ*0/MET15 ura3*Δ*0/ura3*Δ*0*) strains of the budding yeast *Saccharomyces cerevisiae* were used in this study. The high copy yeast genomic library (ATCC No. 37323) was used for genomic library screenings. Yeast transformations were performed by the standard LiAc method. Unless indicated otherwise, all experiments were performed on Yeast Nitrogen Base (YNB, 2% Glucose) media supplemented with appropriate amino acids and bases.

For yeast expression experiments, the genes that reside within the original YEp13 genomic clones that caused resistance against Doxorubicin, were each cloned separately into the pAG426-GPD plasmid (Addgene) and then expressed under control of the GPD promoter, except for PDR5 plasmid, which was obtained from Prof. Dr. Wenjun Guan (Zhejiang University, China). For plasmid isolations, yeast cells were predigested by lyticase (5u/ml) for 30 minutes in Tris-EDTA (TE) buffer before the isolation and plasmids were isolated from yeast cells by using GeneJET Plasmid Miniprep kit (Thermo-Molecular Biology) as described by the manufacturer. The isolated plasmids were amplified in *E*. *coli* DH5α cells and sequenced by using a pair of vector-specific primers at IzTech Biotechnology Center (Izmir). Doxorubicin was purchased from SABA pharmaceuticals (Cat No.: 8699511796063 /Turkey).

### Gradient spot assays

Petri plates with a continuous gradient of a drug was described by Szybalski and Bryson (1952). Briefly, two layers of agar were poured into a square petri dish. The bottom layer contained normal medium and the plate was propped up slightly for agar to cover the entire bottom. When the agar was solidified, the dish was placed in a horizontal position and doxorubicin harbouring medium (50 ml) was added on top of the plate. Downward diffusion of doxorubicin resulted in its dilution proportional to the thickness of the agar layers and established a concentration gradient changing from approximately 0 μM on one side to 500 μM on the other [24].

The WT BY4741 and BY4743 strains carrying the plasmids were grown overnight diluted by growth media and incubated 3h to obtain exponentially growing cells. Cells were washed with dH_2_O, diluted to OD_600_ 0.02 and 5 μl of cell solution was transferred to each spot. Plates were photographed after three days of incubation at 30°C.

### RNA isolation and real-time PCR analysis

Total RNA samples from exponentially growing yeast cells were isolated using the RNeasy Mini Kit (Qiagen). Genomic DNA contaminations were removed by DNase treatment (Dnase RQ1, Promega). cDNA synthesis was performed using the First Strand cDNA Synthesis Kit (Fermentas) according to the manufacturer’s instructions. The cDNAs were used as templates to amplify internal parts of the selected genes. *ACT1* gene was used as an internal control. Real-time PCR assays were performed with IQ5 real-time PCR system (BIO-RAD).

### Microarray analysis

Concentrations and purity of RNA samples were determined by measuring their absorbances at 260/280nm, using a nanodrop spectrophotometer (Thermo Scientific), and the quality of RNAs were determined by Agilent RNA 6000 Nano Kit in Agilent Bioanalyzer.

Total RNA and spike-in mixes were prepared by mixing minimum 100ng of total RNA and spike-in kit for each qualified RNA sample. Total RNA sample was resuspended in nuclease-free water to obtain minimum of 50ng/μl RNA. The spike-in solution and T7 primer mix was added on diluted RNA samples and denaturation was performed by incubating the mix at 65^°^C for 10 minutes. cDNA master mix was prepared and added on each RNA sample mix. The mix was then incubated at 40^°^C for 2 hours; 70^°^C for 10 minutes and on ice for 5 minutes, respectively. The Cyanine-3-labeled and amplified RNA samples (cRNA) were purified and quantification was performed by using Nanodrop spectrophotometer (Thermo Scientific).

For hybridization, cRNA samples were incubated with a fragmentation buffer and gene expression blocking agent at 60^°^C for 30 minutes. The fragmented samples were loaded on arrays and the array slides were placed in a hybridization oven and the hybridization reaction was performed at 65^°^C for 18 hours. After the hybridization step, the samples were washed with a washing solution. The washing step was performed at room temperature for the first and the second washes. The third wash was performed at 37^°^C. After the washing step, the slides were scanned and signal intensities were obtained by a feature extraction program.

### Statistical analysis

Student’s T-test was used for Real-time PCR analysis and fold-change analysis was used for microarray analysis data. P–value 0.05 was chosen as the significance level (p< 0.05) for statistical analysis.

## Results and Discussion

### Screening for the genes that show resistance to doxorubicin

**Fig 1:**
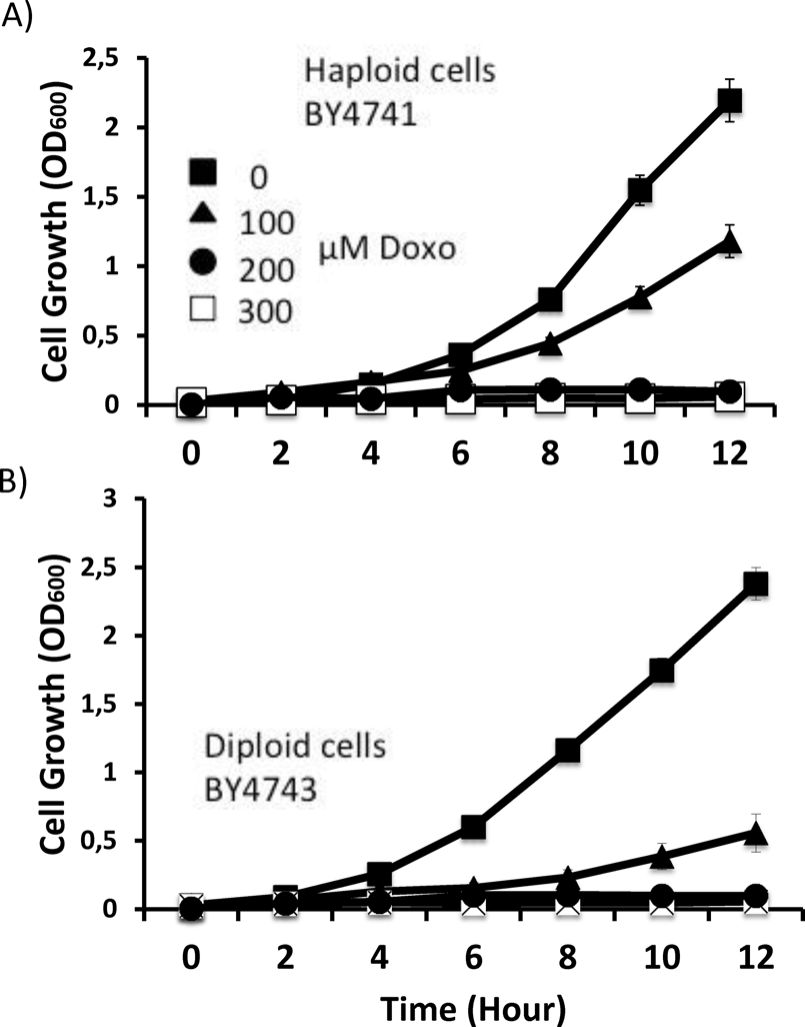
Growth curves. Wild type (**A)** haploid and (**B)** diploid yeast strains on 0 μM, 100 μM, 200 μM and 300 μM doxorubicin media. Error bars represent SD of the means for triplicate determinations.

In order to identify genes that confer resistance to doxorubicin, we first determined the toxic drug concentrations for our strains. We analyzed cell growth rates in different drug concentrations in liquid media for both haploid (BY4741) and diploid (BY4743) wild type cells. As seen in Fig 1, the growth of both strains was completely inhibited in the presence of 200 μM or higher amounts of doxorubicin. Next, we transformed wild type (BY4741) cells with a 2μ-based genomic expression library and isolated 6 transformants that could grow in the presence of 500 μM of doxorubicin on solid media. By using a high concentration of doxorubicin for screening, we aimed to find genes that help cells tolerate very toxic levels of the drug. Plasmids from the transformants were isolated and amplified in *E*. *coli* and used for the re-transformation of fresh wild type cells for confirmation purposes. All the isolated plasmids conferred resistance to the new cells, thus, we confirmed that the resistance observed in the original transformants were provided by the plasmids.

**Fig 2:**
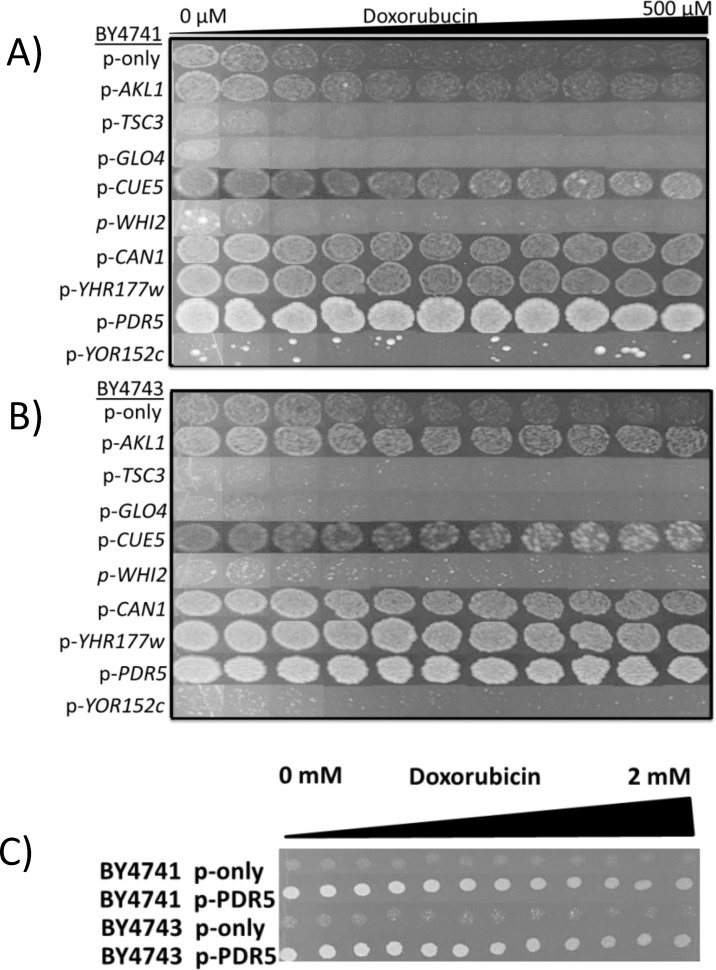
Spotting assays for overexpression analyses. Candidate genes that play role in doxorubicin resistance were cloned into pAG426-GPD plasmid and expressed in **(A)** haploid and **(B)** diploid wild type strains in the presence of doxorubucin. **(C)** Overexpression of the *PDR5* gene could provide resistance to 2 mM of doxorubucin.

The nucleotide sequences of the expression cassettes in each plasmid were determined. Sequence analyses yielded nine intact genes (Table 1). In order to find out which one of these genes provided resistance, they were all cloned onto the pAG426-GDP plasmid individually and expressed in haploid (BY4741) and diploid (BY4743) wild type cells. As seen in Fig 2A and 2B, only expression of *AKL1*, *CUE5*, *CAN1*, *YHR177w* and *PDR5* genes provided resistance to doxorubicin. Particularly, cells overexpressing *PDR*5 gene were highly resistant to the drug and able to grow in the presence of 2 mM of doxorubicin (Fig 2C), which is the highest dose that could be tolerated by yeast cells as of our knowledge.

**Table 1:** Genomic sequences that were responsible for doxorubicin resistance.

Colony No	Chromosome Information	Covered Genes
1	Chromosome XV Coordinates 407059 bp to 411911 bp	GLO4, CUE5, part of WHI2
2	Chromosome V Coordinates 30280 bp to 33605 bp	CAN1
3	Chromosome II Coordinates 346000 bp to 366000	TSC3, AKL1
4	Chromosome VII Coordinates 445000 bp to 465000 bp	YHR177w
5	Chromosome XV Coordinates 607000 bp to 627000 bp	YOR152C, PDR5

**Fig 3:**
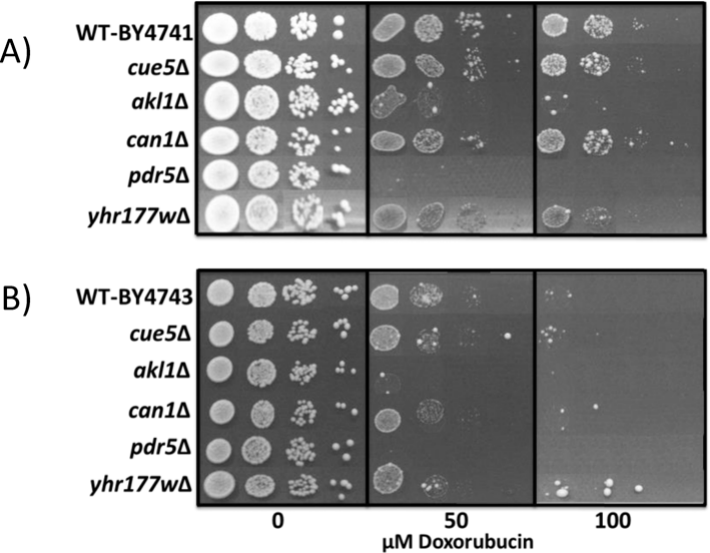
Spotting assays for sensitivity analyses. **(A)** Haploid and **(B)** diploid deletion mutants of the candidate genes were analyzed by a spotting assay for their doxorubicin sensitivity. Serial dilutions (5 μl) (OD_600_ 0.2, 0.02, 0.002 and 0.0002) for each strain were used for the sensitivity analyses.

As the next step, we analyzed the deletion mutants of *AKL1*, *CUE5*, *CAN1*, *YHR177w* and *PDR5* genes in both haploid and diploid backgrounds to identify whether they were sensitive to doxorubicin (Fig 3A and 3B). In both backgrounds, *pdr*5Δ mutants were the most sensitive cells and their growth was inhibited by 50 μM of doxorubicin. In addition to *pdr*5Δ mutants, *akl1*Δ cells were also more sensitive to doxorubicin when compared to wild type cells, however the rest of the mutants (*cue5*Δ, *can1*Δ and *yhr177w*Δ) showed a growth pattern similar to that of wild type cells.

Pdr5p is a member of the ATP-binding cassette family of transporters and mediates resistance to many xenobiotics such as mutagens, antifungals and steroids [25,26]. In addition to drug stress, it is also involved in cation resistance [27], and lipid transport in yeast cells [28]. Pdr5 resembles (orthologous) mammalian MDR1 which is a major factor in tumor resistance [29]. Regulation of *PDR5* is controlled by transcription factors *PDR1* and *PDR3* and deletion of these transcription factors leads to loss of *PDR5* expression, while gain of function mutations in pdr1/3 leads to over expression of this protein [30–32].

Being an efflux pump, *PDR5* gene has previously shown to be associated with doxorubicin resistance. Golin et. al. showed that *pdr5*Δ mutants could not grow at 50μM doxorubicin [33] and Rogers et. al. showed that *pdr5*Δ mutants are sensitive to doxorubicin [34]. Kolaczkowski et al (1996) showed that *pdr1-3* mutants, that overexpress *PDR5*, are doxorubicin resistant, while the *pdr5*Δ mutant is sensitive to the drug [35]. A more recent study has shown that *pdr1-3* mutation leads to upregulation of about twenty-five other genes in addition to *PDR5* [36], which may also affect the resistance level in *pdr1-3* mutants. Here, we showed that not only *pdr5*Δ mutants were doxorubucin sensitive but also *PDR5* overexpression from a plasmid increased doxorubicin resistance, which supports the findings from previous studies [33–35].

**Fig 4:**
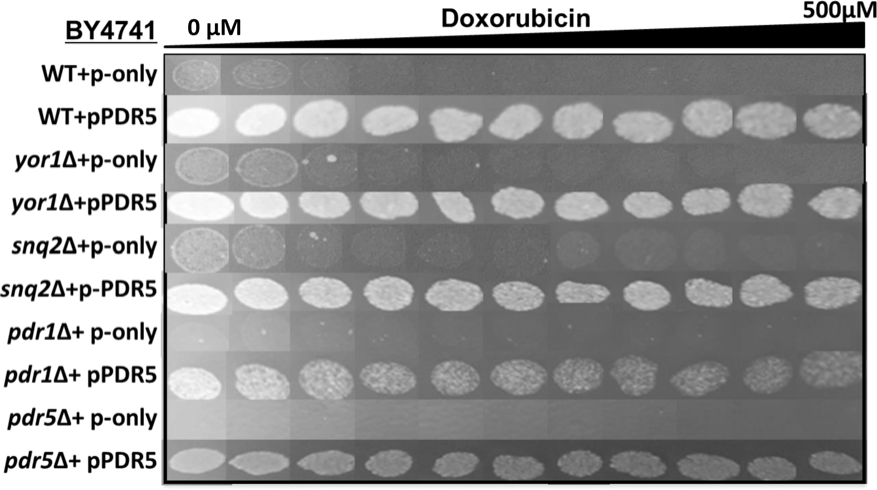
*PDR5* overexpression analyses in *yor1*∆, *snq2*∆ and *pdr1*∆ mutants. *PDR5* gene was overexpressed in wild type, *yor1*Δ, *snq2*Δ, *pdr1*Δ, and *pdr5*Δ cells and growth rates were determined by a spotting assay in the presence of doxorubicin gradient.

*PDR5*, *SNQ2* and *YOR1* are known to be major determinants of multidrug resistance in yeast and they all are controlled by *PDR1* [30,37,38]. In order to check if *PDR5* overexpression may lead to resistance to doxorubucin in the absence of these genes, we overexpressed *PDR5* in *yor1*∆, *snq2*∆ and *pdr1*∆ mutants and analyzed them by a spotting assay (Fig 4). In addition to doxorubicin, transformants were also tested on clatrimazole, an inhibitor of ergosterol synthesis in fungi [39], and cerulenin, an inhibitor of fatty acid synthesis [40], since both are known to be substrates of Pdr5 (S1 Fig). *PDR5* overexpression made these cells resistant to all three drugs. The *pdr1*Δ strain was more sensitive to the drugs compared to the wild type cells, whereas s*nq2*Δ and *yor1*Δ mutants showed no sensitivity. Cerulenin was very toxic to cells and only cells that carried *PDR5* plasmid were able to survive in the presence of this drug even at the minimal concentrations (S1 Fig). We also noticed that *PDR5* overexpressing *pdr1*Δ cells were less resistant to the drugs compared to *PDR5* overexpressing wild type cells. On the other hand, y*or1*Δ mutants were previously shown to be sensitive to doxorubicin [34], however we did not observe this phenotype under our experimental conditions.

**Fig 5:**
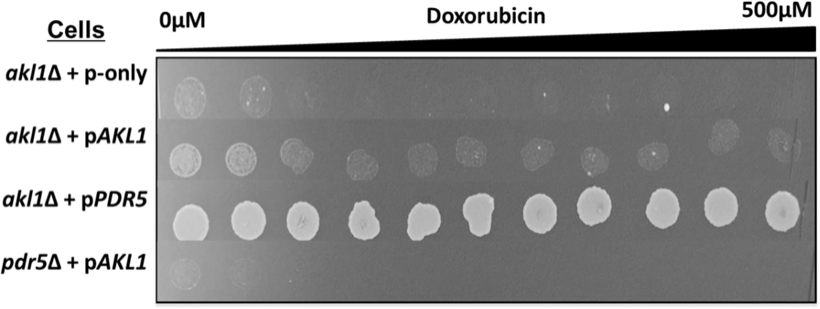
*AKL1* overexpression analyses in *pdr5*Δ mutants. *AKL1* was overexpressed in haploid *pdr5*Δ mutants and doxorubicin resistance was tested by a spotting assay.

Another gene whose overexpression provided resistance and deletion rendered cells to doxorubicin was *AKL1*. Akl1, which is a serine-threonine protein kinase involved in endocytosis and actin cytoskeleton organization [41], has previously been linked to doxorubicin resistance by others [14]. Sla1/Pan1/End3 complex is involved in endocytosis and affected by Akl1 overexpression [20]. Akl1 phosphorylates Pan1p and leads to dissociation of the yeast Sla1/Pan1/End3 complex, which regulates the internalization step of endocytosis [42]. Dissociation of the complex subsequently results in inhibition of endocytosis. Reduction in endocytosis did not affect doxorubicin accumulation significantly [20], and only very few drugs have been shown to enter cells through endocytosis [29]. Therefore, resistance to doxorubicin provided by Akl1p overexpression may be through mechanisms other than reduced endocytosis. However, reduction in endocytosis may have indirect effects on doxorubucin resistance, because *end4* endocytosis mutants accumulate Pdr5p at the plasma membrane [35,43]. To test if doxorubicin resistance in *AKL1* overexpressing strain is somehow affected by the presence of Pdr5, we overexpressed *AKL1* in *pdr5*Δ mutants and spotted cells in the presence of doxorubicin (Fig 5) and other drugs (S2 Fig). Our results indicated that *AKL1* overexpression in *pdr5*Δ mutants did not make cells resistant to doxorubicin or to other drugs, which suggested that *PDR5* was required for Akl1 action. It is likely that *AKL1* overexpression may act through Pdr5 stabilization in plasma membrane. Overexpression of AAK1, which is the human orthologue of *AKL1*, also causes doxorubicin resistance in Hela cells [20], suggesting that the role of *AKL1* in doxorubicin resistance is conserved in higher eukaryotes, but the mechanisms of resistance are not clear yet.

*CAN1*, *YHR177W* and *CUE5* are the other genes obtained from genomic library screening, of which their overexpression made cells resistance to doxorubicin, but their deletion mutants did not show any sensitivity. Can1p is a plasma membrane arginine permease, arginine-H^+^ symporter, which is exclusively associated with lipid rafts [44]. Possible doxorubicin resistance mechanism for *CAN1* may be upregulation of filamentous growth due to its location in ergosterol-rich domains of the plasma membrane that harbors several proteins required for filamentous growth [45].

*YHR177w* is a putative transcription factor with a WOPR domain, of which its overexpression causes either cell cycle delay or arrest [46]. Proteins with WOPR domains are important in pathogenesis [46] and *YHR177w* overexpression leads to invasive growth (pseudohyphae formation) in yeast [47]. Therefore, *YHR177w* overexpression may also cause doxorubicin resistance through activation of invasive growth.

Cue5p functions as ubiquitin-Atg8p adaptor in ubiquitin-dependent autophagy [48,49] and it is role in drug resistance is not clear.

### Expression patterns of resistance genes in response to doxorubicin treatment

**Fig 6:**
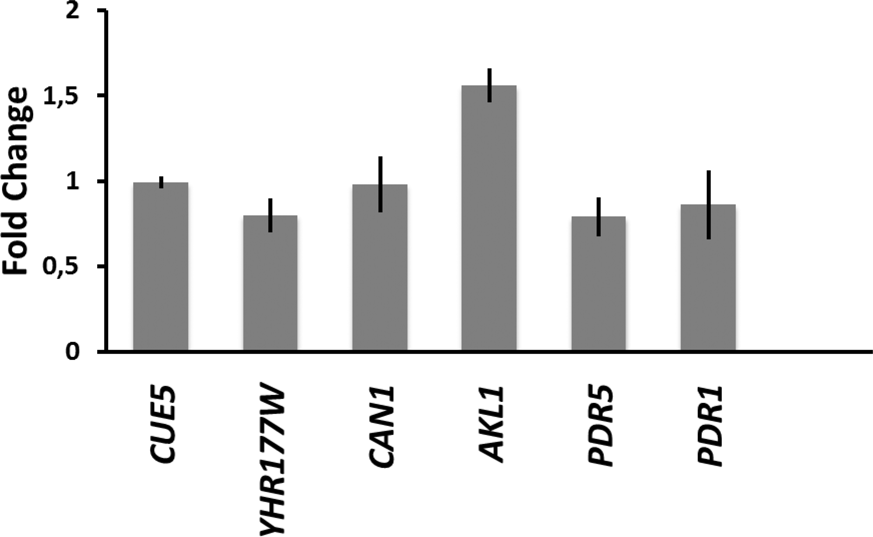
Real-Time PCR analyses of the genes that cause resistance to doxorubicin. The expression levels for genes were normalized by their untreated counterparts. Each sample was assayed at least 3 times and error bars show the SD of the means. *ACT1* gene was used as the internal control.

Screening analyses showed that the overexpression of *PDR5*, *AKL1*, *CAN1*, *YHR177W* and *CUE5* from a plasmid provided doxorubicin resistance, however it is not known whether these genes are upregulated or not in the presence of doxorubicin. In order to study expression patterns of these genes, we incubated cells with a sublethal dose of doxorubicin (80 μM) for two hours and determined the transcript levels of each gene by a Real-Time PCR approach (Fig 6). Interestingly, expression of only *AKL1* increased slightly (1.5-fold) (p = 0,25) and the rest of the genes did not physiologically respond to the drug. Expression analyses also showed that *PDR1*, a known activator of *PDR5*, expression was not activated by doxorubicin treatment. Thus, these genes were not transcriptionally responsive to doxorubicin and provided resistance only if they were expressed ectopically. On the other hand, doxorubicin sensitivity of *pdr*5Δ and *akl1*Δ mutants suggested a possible role for these genes in doxorubicin resistance with their basal expression levels.

### Global expression profiling for doxorubicin response

To further evaluate the mechanisms of doxorubicin resistance and toxicity, we analyzed the changes in the global gene expression profile of yeast cells after doxorubicin exposure.

The genes showing more than 2-fold changes (p<0.05) are listed in S1 Table. Our microarray results also showed that doxorubicin resistance genes were not upregulated in response to the drug treatment and were consistent with the qPCR data. Out of approximately 6200 yeast genes, 211 of them were significantly upregulated, while 148 genes were downregulated. We categorized these genes by functional MIPS classification (Tables 2 and 3) to highlight the pathways that play role in doxorubicin tolerance and toxicity.

**Table 2:** MIPS functional classification of >2-fold upregulated genes in response to Doxorubicin treatment (p value cutoff: 0.01).

Category	p-value	In Category from Cluster	k	f
DNA restriction [10.01.09.03]	1.865e-05	AI1 AI2 AI4 MMS4 MRE11	5	12
detoxification [32.07]	0.003305	ADH5 FZF1 TPO2 AZR1 YHK8 PDR18 PNT1 SVS1	8	80
meiosis I [10.03.02.01]	0.006791	REC104 MRE11 NDJ1	3	13
DNA integration [10.01.05.03.05]	0.009102	AI1 AI4	2	5

**Table 3:** MIPS functional classification of >2-fold downregulated genes in response to Doxorubicin treatment (p value cutoff: 0.01).

Category	p-value	In Category from Cluster	k	f
ion transport [20.01.01]	0.0003473	FIT1 FIT2 FIT3	3	7
sugar transport [20.01.03.01]	0.004537	HXT15 HXT13 HXT16 HXT17	4	31
proton driven antiporter [20.03.02.03.01]	0.00948	KHA1 ATO2	2	7

Funspec analysis [50] of the upregulated genes showed that paths related to transport dependent-detoxification systems and DNA metabolism such as DNA restriction, integration and recombination were upregulated in the presence of doxorubicin (Table 2).

Among the transporters (Table 2) polyamine transporter *TPO2*, azole resistance gene *AZR1*, drug:H(+) antiporter YHK8, pleotropic drug resistance gene *PDR18* and membrane proteins Pnt1 and Svs1 were upregulated in response to doxorubicin. Pdr18, besides being a membrane transporter, can affect the membrane sterol composition and lead to MDR phenotype [51]. Among the upregulated genes *MRE1* (meiotic recombination) and *MMS4* (Methyl Methane Sulfonate Sensitivity) both function in DNA repair [52,53], and are related to doxorubicin sensitivity [15,54].

When we functionally categorized the down-regulated genes in response to doxorubicin treatment, only a small fraction of genes were clustered (Table 3).

Mainly, expression of iron transporters (*FIT1*, *FIT2* and *FIT3*), sugar transporters (*HXT15*, *HXT13*, *HXT16* and *HXT17*) and proton driven antiporters (*KHA1* and *ATO2*) were inhibited by doxorubicin.

Yeast responds to iron limitations by activating Aft1 transcription factor and expressing *FIT1*, *FIT2* and *FIT3* genes. These genes encode for mannoproteins that are incorporated into the cell wall and play roles in retention of siderophore-iron in the cell wall [55]. Decreasing iron levels by inhibition of *FIT1*, *FIT2* and *FIT3* genes might be a good defense system for doxorubicin toxicity, since iron plays role in doxorubicin cytotoxicity by producing ROS [4]. Sugar transporters that were downregulated by doxorubicin are all low affinity hexose transporters and their roles in drug response is not known.

Ion transporters *KHA1* and *ATO2* play role as K(+)/H(+) antiporter and ammonia extruder, respectively [56,57]. Inhibition of *KHA1* may lead to accumulation of K(+) and disruption of vacuole membrane potential, which might be important in doxorubicin transport/defense. However, resistance to doxorubicin decreases in *kha1*∆ mutants [54]. Thus, the exact role of *KHA1* in doxorubicin resistance is not clear. Similarly, possible benefits that could be gained by lowering the transcript levels of *ATO2*, ammonia transporter, are not clear.

Our microarray and real-time PCR results were consistent with each other. They both confirmed that none of the genes obtained from genomic library screenings were upregulated by doxorubicin treatment. The genomic DNA library used in this work was a high copy number (2 μ) library and supposedly genes on the plasmids were expressed at high levels. We observed that *PDR5* mRNA level was 7-fold higher than that of empty vector carrying transformants (S3 Fig) (p = 0,039) and that was apparently enough for cells to tolerate 2mM doxorubicin.

Seemingly, doxorubicin did not activate the transcriptional machinery required for the expression of *PDR5*, *CAN1*, *YHR177W* and *CUE5* genes since their mRNA levels did not change much upon the treatment. A specific support for the transcriptional inertness of these genes was the unaffected level of *PDR1*, an activator of *PDR5* gene (Fig 6). In addition to Pdr1, transcription factors Pdr3, Yap1, and Mig3 also play roles in expression of *PDR5* in response to drug/chemical stress exposure [58], however, their mRNA levels were not increased significantly by the doxorubicin treatment (S1 Table). Thus, even though *PDR5* played a major role in doxorubicin tolerance, the transcription factors that regulate it were not activated by doxorubicin treatment.

## Conclusion

In this study, we screened a yeast genomic DNA library to identify genes that are responsible for doxorubicin resistance and found that *PDR5* was the primary gene that played role in doxorubicin tolerance. Our screen also pointed out roles of other genes such as, *AKL1*, *CAN1*, *YHR177W* and *CUE5* in protecting cells from doxorubicin toxicity. We also showed that transcriptional regulation of these genes was not dependent on doxorubicin treatment, however, overexpression of them make *S*. *cerevisiae* cells resistant to high doses of doxorubicin.

Additionally, we analyzed the global expression profile of yeast cells after doxorubicin treatment and highlighted the genes and paths that might be important in doxorubicin tolerance and toxicity. Our results showed that membrane transporters

and DNA metabolism genes are upregulated in the presence of doxorubicin and these genes may function in doxorubicin detoxification/tolerance processes.

When we consider the genes whose overexpression caused doxorubicin resistance, excluding Cue5, their common effect seemed to be the change in membrane asymmetry. Thus, changing the composition of the cell membrane could be a common response of cells to high doxorubicin levels.

## Supporting Information

S1 Fig Spotting assays for PDR5 overexpression. *PDR5* was cloned and expressed in wild type, *yor1*Δ, *snq2*Δ, *pdr1*Δ, and *pdr5*Δ cells. Spotting assays were performed on (A) clatrimazole and (B) cerulenin.
(TIFF)

S2 Fig Spotting assays for *AKL1* overexpression. *AKL1* was cloned and expressed in *akl1*Δ and *pdr5*Δ. Spotting assays were performed on (A) clatrimazole and (B) cerulenin.
(TIFF)

S3 Fig Real-time PCR analyses for *PDR5* overexpression. *PDR5* transcript analyses in haploid and diploid wild-type strains that overexpress *PDR5*.
(TIFF)

S1 Table List of the genes that were up or down-regulated by 2-fold or more in response to doxorubicin treatment. (XLSX)
