# Supplementary figures and images for: High-Copy Overexpression Screening Reveals PDR5 as the Main Doxorubicin Resistance Gene in Yeast
Source: PLoS One. 2015 Dec 21;10(12):e0145108. doi: 10.1371/journal.pone.0145108 (PMC4687100; doi:10.1371/journal.pone.0145108)

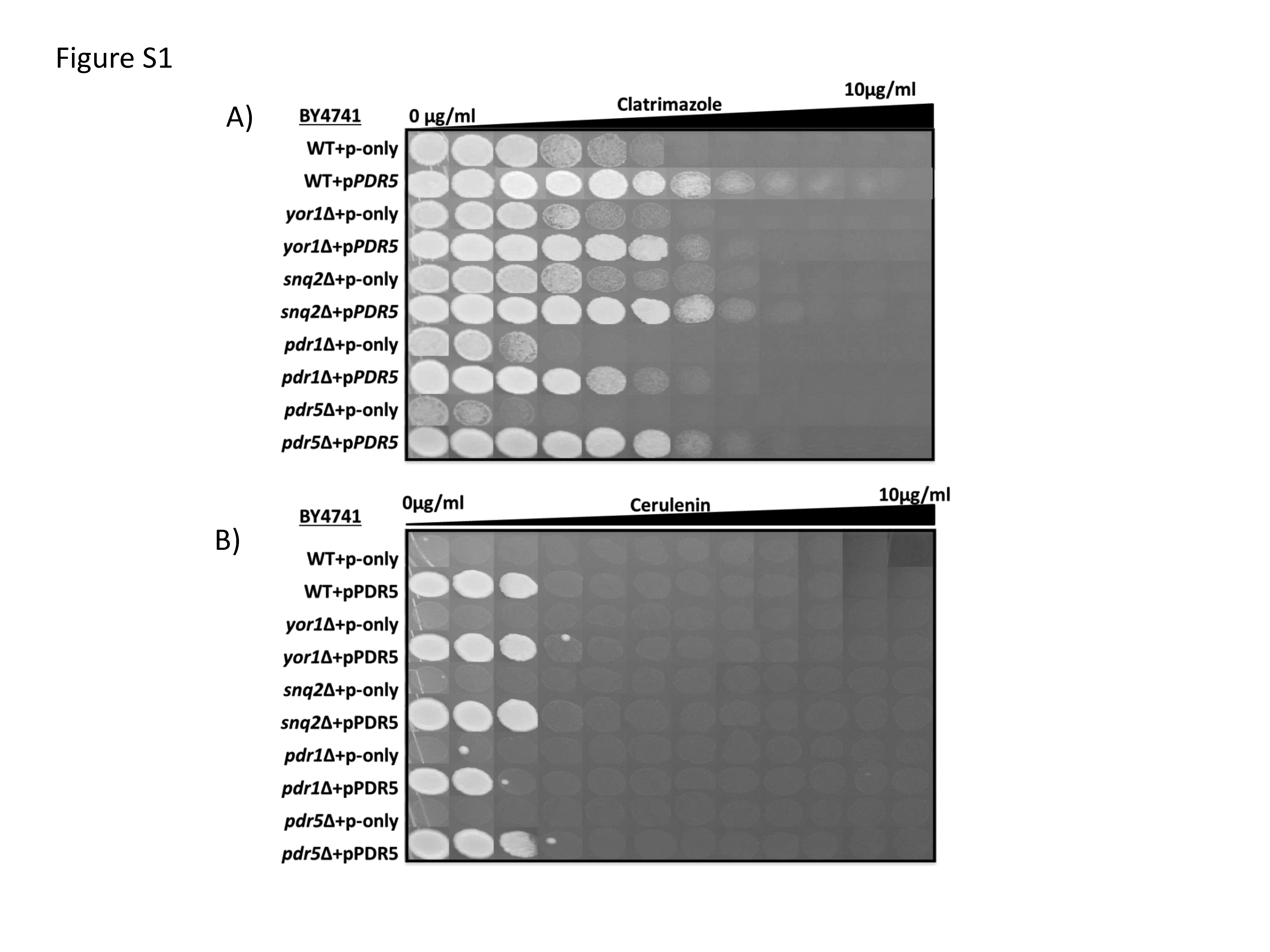

Supplement: S1 Fig — PDR5 was cloned and expressed in wild type, yor1Δ, snq2Δ, pdr1Δ, and pdr5Δ cells. Spotting assays were performed on (A) clatrimazole and (B) cerulenin. (TIFF) [file pone.0145108.s001.tiff]

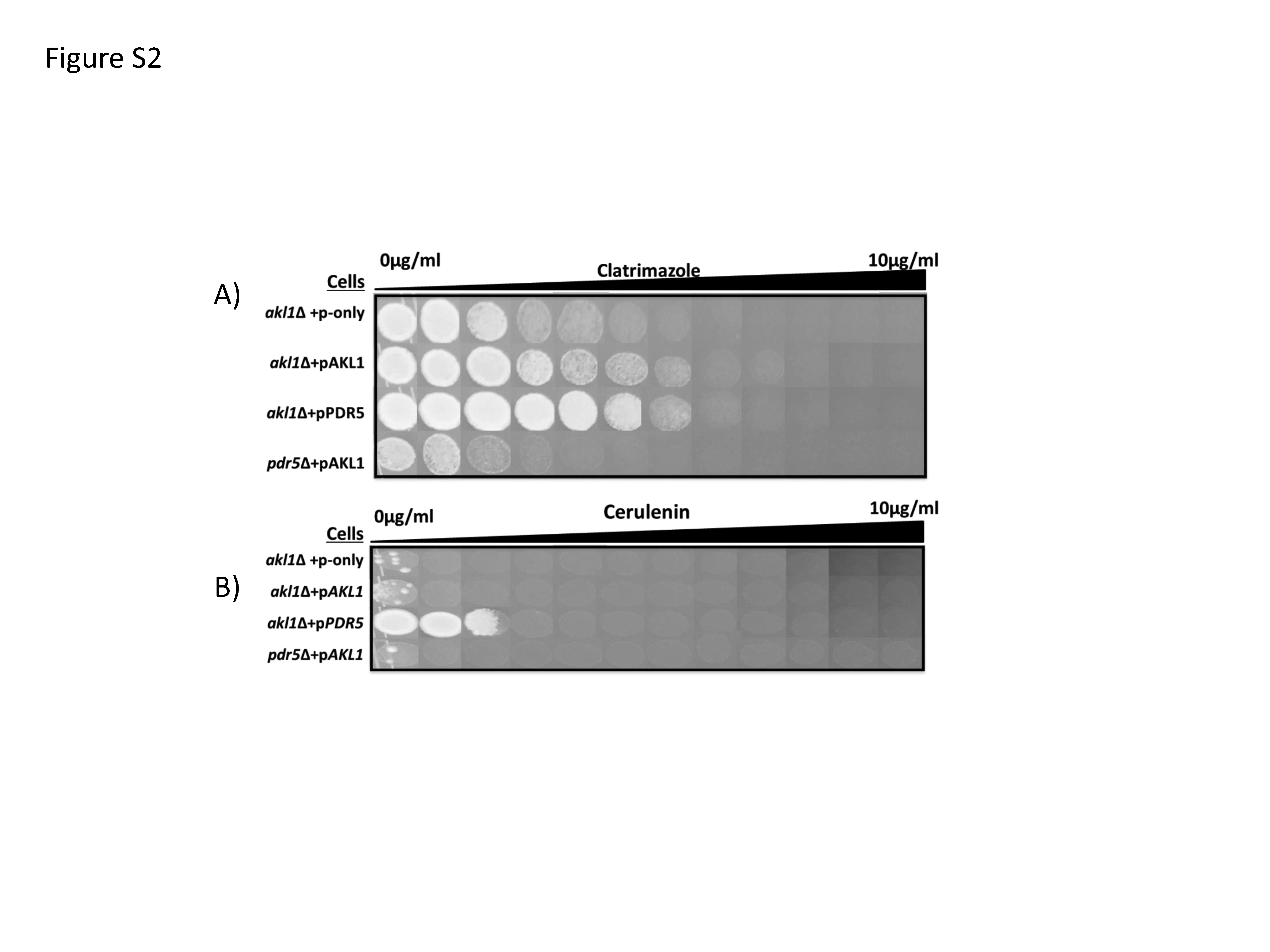

Supplement: S2 Fig — AKL1 was cloned and expressed in akl1Δ and pdr5Δ. Spotting assays were performed on (A) clatrimazole and (B) cerulenin. (TIFF) [file pone.0145108.s002.tiff]

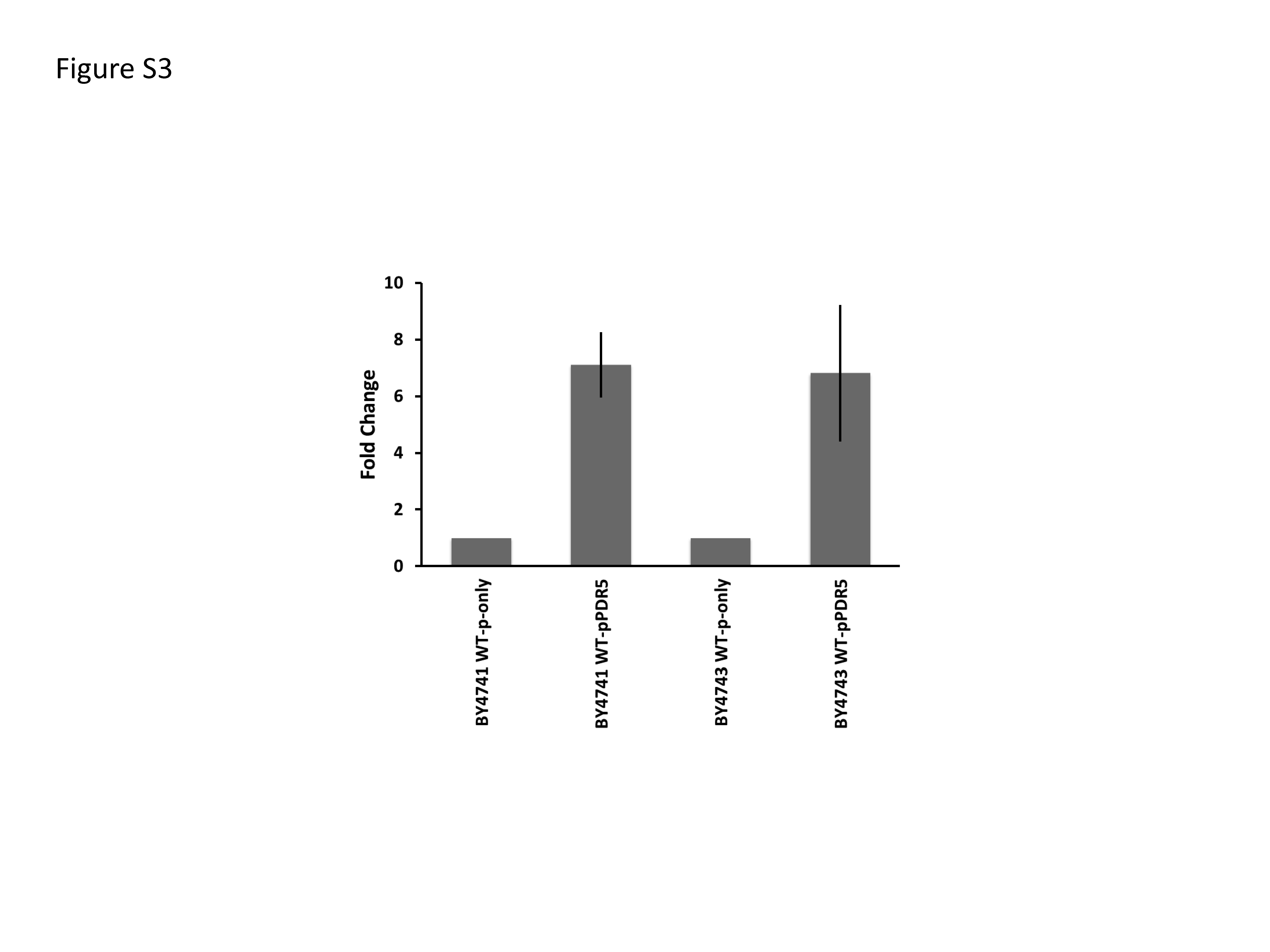

Supplement: S3 Fig — PDR5 transcript analyses in haploid and diploid wild-type strains that overexpress PDR5. (TIFF) [file pone.0145108.s003.tiff]
